# Editorial: Recent advances in crop diseases associated with plant vascular-colonizing bacteria

**DOI:** 10.3389/fpls.2023.1171973

**Published:** 2023-03-30

**Authors:** Wei Wei, Essaid Ait Barka, Ales Eichmeier

**Affiliations:** ^1^ Beltsville Agricultural Research Center, Molecular Plant Pathology Laboratory, Agricultural Research Service, United States Department of Agriculture, Beltsville, MD, United States; ^2^ Unité de Recherche Résistance Induite et Bio-Protection des Plantes-EA 4707 – USC INRAe1488, Université de Reims Champagne-Ardenne, Reims, France; ^3^ Mendeleum – Institute of Genetics, Mendel University in Brno, Brno, Czechia

**Keywords:** plant vascular system, Huanglongbing (HLB), *Candidatus* Liberibacter asiaticus, citrus, black rot disease, *Xanthomonas campestris*

The plant vascular system is a vital part of healthy plants and is composed of two primary tissue types: phloem and xylem. The phloem transports carbohydrates from the source leaves to where they are needed for growth and development, while the xylem delivers water and mineral ions from the roots to the rest of the plant. These two types of vascular tissues form a complex and essential network that supports the overall functioning of the plants. Despite its importance, the plant vascular system is vulnerable to colonization by phytopathogenic bacteria, including wall-less Mollicutes, walled phloem-limited bacteria, walled xylem-restricted bacteria, and other bacteria that invade the plant through different pathways. The infection of plants by these bacteria can often lead to stunted growth, wilting, necrosis, flower deformation, tumors/galls, and even plant death, resulting in serious economic losses to commercial crops and ornamental trees.

In this Research Topic, we aim to highlight the current state of research and understanding of the mechanisms by which plant vascular-colonizing bacteria cause diseases. The contributing articles delve into Huanglongbing (HLB), which is caused by the bacterium *Candidatus* Liberibacter asiaticus (CLas), and black rot disease caused by the bacterium *Xanthomonas campestris* pv. *campestris*.

Citrus crops are important in many regions, and their production is threatened by HLB disease caused by CLas, which seriously affects the livelihoods of farmers and the citrus industry. The study by Huang et al. sheds light on the population diversity of CLas strains in citrus trees. The authors used a prophage gene-based typing system and machine learning tools to establish two CLas geno-groups and found evidence of at least two different introductions of CLas strains into Mexico. In addition, the study raises important questions about the origin, transmission, and management of HLB, which has important implications for citrus growers and researchers around the world and could help to develop new management strategies against HLB, such as the use of resistant varieties, the development of early detection methods, or the deployment of natural enemies of the insect vector. The study also highlights the importance of international cooperation in addressing the HLB challenge. For example, the findings of this study demonstrated the close relatedness between CLas strains in Mexico and the United States, underscoring the need for coordinated efforts to prevent the disease from spreading across borders.

Cifuentes-Arenas et al. investigated the effects of HLB on the yield and fruit quality of five-year-old Sicilian lemon trees in São Paulo State, southeastern Brazil, over a five-year period (2017-2021). The study found that the symptoms of HLB increased over time, and it took an estimated 10 years for symptoms to occupy over 90% of the tree canopy. Compared to healthy trees, trees with symptoms on 20%, 50%, and 80% of their canopy experienced significant reductions in both yield and fruit quality, with an average reduction in production of 18%, 38%, and 53% respectively. Additionally, the fruits produced by the symptomatic branches of lemons were lighter, and the number of dropped fruits was not related to the symptom severity. The study also observed that the flushing on symptomatic branches started earlier compared to healthy branches of lemon and orange trees. This early flushing is likely an attempt by the tree to compensate for the loss of photosynthetic capacity due to HLB infection, but it also leads to a faster progression of symptoms and a greater decline in production.

Another study conducted by Carvalho et al. provides important insights into the potential of late-season sweet orange selections for diversifying citrus orchards and mitigating the risks of pest and disease outbreaks in humid subtropical regions. In a long-term field experiment conducted in northwestern Paraná State, Brazil, the authors assessed the horticultural characteristics of 19 late-season sweet orange selections under endemic conditions of citrus canker and HLB. The findings demonstrate the importance of diversifying citrus orchards through the cultivation of resistant and late-season sweet orange selections, which can help increase productivity and profitability. The study also highlights the need for further research to evaluate the performance of these selections under different environmental conditions and to develop sustainable methods to control citrus canker and HLB.

However, more research is needed to fully understand the underlying mechanisms of HLB and to develop effective management strategies. This includes studying genetic and ecological factors that influence the spread and impact of the disease, as well as exploring innovative technologies and methods for controlling insect vectors and reducing the spread of CLas. The research community, policymakers, and industry stakeholders need to work together to support research efforts and implement the best available strategies to manage this devastating disease.

In addition, black rot disease caused by the bacterium *Xanthomonas campestris* pv. *campestris* (Xcc) is a significant problem in *Brassica* crops, such as oilseed rape (*Brassica napus* L.), causing substantial yield losses worldwide. However, our understanding of the molecular mechanisms underlying *Brassica*’s resistance to black rot remains limited. Yang et al. compared the gene expression profiles of two EMS (ethyl methanesulfonate)-mutagenized *B. napus* lines that exhibited contrasting levels of resistance to Xcc with their susceptible progenitor. The findings revealed a steady number of differentially expressed genes (DEGs) in the susceptible line ZS9mXccS-1 at different time points of infection, whereas the resistant line ZS9mXccR-1 showed a gradual increase in DEGs throughout the course of infection. Furthermore, the authors used a weighted gene co-expression network analysis (WGCNA) to identify hub genes involved in immunity. Multiple defense-related hub genes were highlighted, including the cell surface receptor genes CRK11 and BIR1, and the associated downstream regulatory genes WRKY11 and PBL30. Additionally, the KEGG analysis of DEGs belonging to two distinct co-expression modules revealed enriched pathways associated with defense, including Ca^2+^ signaling, receptor-mediated immunity, and phytohormone balance. These results provide new insights into the molecular mechanisms of resistance to black rot disease in *Brassica* crops. The findings of this study might facilitate the development of improved breeding strategies and the identification of key genes and pathways involved in resistance to Xcc in *Brassica* crops.

The published articles in this Research Topic not only shed light on the mechanisms of disease caused by vascular colonizing bacteria in plants but also address the challenges and opportunities facing this area of research. As a result, these findings are expected to provide valuable guidance for future research and policy decisions. These pathogens have a significant impact on agriculture, horticulture, forestry, and natural ecosystems, so it is important that researchers, scientists, stakeholders, and policymakers work together to address these challenges.

## Author contributions

The authors listed have made a substantial, direct, and intellectual contribution to the work and approved it for publication.

